# Making of a single solid-state nanopore on the wall of fused silica capillary

**DOI:** 10.1098/rsos.171633

**Published:** 2018-06-06

**Authors:** Fang Fang, Yan-Qin He, Li Tian, Yun-Yun Li, Zhi-Yong Wu

**Affiliations:** Research Center for Analytical Sciences, Department of Chemistry, College of Sciences, Northeastern University, Shenyang, 110819, People's Republic of China

**Keywords:** single solid-state nanopore, fused silica capillary, HF etching, ion concentration polarization, rectification, translocation

## Abstract

A channel of nanometre size is an important platform for nanofluidic investigations. In this work, we show that a single solid nanopore can be generated by hydrogen fluoride etching of the outside wall of commercially available fused silica capillary. The geometry, size and the singleness were characterized by various means, including scanning electron microscope, electrolyte conductance measurement and a fluorescent microscope. The generation principle is also discussed. The nanopore thus generated features in low aspect ratio and exhibits typical nanofluidic effects such as ion concentration polarization, rectification and molecular translocation.

## 1. Introduction

Nanofluidic interface demonstrates many unique features, such as ion concentration polarization (ICP), owing to the overlap of an electrical double layer and ion rectification owing to asymmetry in shape or charge distribution [1,2]. An interface with a single nanopore/channel has drawn particular attention owing to its discrimination capability with ionic conductance pulses caused by the translocation of molecules or nanoparticles [3]. Various single nanopore-based applications have been developed, such as DNA sequencing [4,5], biosensors [6,7], molecular interactions [8,9], and single molecule mass spectrometry of polymers [10]. Mainly two types of single nanopore can be recognized in the reported works. One is a soft or bio-nanopore established by assembling of a single pore protein molecule on the lipid membrane of approximately 4 nm thick [4,11]. The other is a solid (or synthetic, artificial) single nanopore fabricated on inorganic or organic polymer substrates. Solid-state nanopore features in stability and size tuning possibility. Pores with a low aspect ratio show higher discrimination power to translocation events [12,13]. The lowest aspect ratio pores can be fabricated on the graphene substrate of several nanometres [14,15], and the next may be that on SiN substrate of 10–50 nm [9,16].The low aspect ratio single nanopores are usually established on a hybrid system, for example, those on graphene consisted of graphene/SiN /SiO_2_/Si supported with a chamber of 50 by 50 µm [14]. Conical single pores can be prepared by track etching of the polymer substrate following heavy ion irradiation [17]. With a highly focused electron beam, single nanopores can be generated on the graphene substrate, and the size can be tuned by the beam dosing [14,15]. With electron beam lithography and reactive ion etching, single nanopores can be generated on the thin SiN substrate [9,17–19]. With focused ion beam milling, a single nanopore as small as 5 nm can be fabricated on various substrates, such as quartz, silicon and polydimethylsiloxane [20]. Besides the above-mentioned sophisticated fabrication methods, simple and cost-effective methods are especially attractive to those who have limited access to advanced facilities and cleaning room conditions. Glass substrate is cheap, and has unique optical, electrical and chemical properties. Several pipette- type single nanopore preparation methods have been developed based on the pulled tip of a glass tube [21–25]. The pore size can be widened by controlled etching or grinding, or shrunken under an electron beam in a transmission electron microscope, as demonstrated by Steinbock *et al.* [26]. Zhang *et al*. prepared a conical single nanopore at a glass tube terminal with the aid of a Pt wire nanotip [27]. Based on the conical nanotip interface of glass, Long and Ying's group realized single molecular detection [28,29] and manipulation of single particles [30,31]. To date, mainly high aspect ratio single nanopores (electronic supplementary material, figures S1A and S1B) are demonstrated with the glass tube.

Early in 1997, Hu *et al*. [32] introduced an interface on the wall of fused silica capillary by hydrogen fluoride (HF) etching, and many applications have been developed using this interface as an ion conductive joint and/or a size filter [33,34]. In 2012, we first noted that both small and macromolecules could electrokinetically stack at the interface, and this was well explained by the ICP effect [35]. Although the reported works showed that there must exist some kind of pore of molecular size on this type of interface, knowledge about the shape, size and number of pore(s) remained relatively poor up to now.

In this work, we reveal for the first time, to our knowledge, that by controlled voltage-assisted HF etching, a low aspect ratio single nanopore on the wall (electronic supplementary material, figure S1C) open to the inside of a glass capillary, rather than on the pulled tip can be obtained.

## 2. Experimental

### 2.1. Materials and reagents

The fused silica capillary (75 or 100 µm i.d. 375 µm o.d.) used in this work was purchased from Yongnian Ruifeng Chromatography Co. Ltd., Hebei, China. The source of major chemicals is as follows: hydrofluoric acid (Shenyang Chemical Co., Liaoning, China), sodium hydroxide (Tianjin Bodi Chemical Co. Ltd., Tianjin, China), potassium chloride (KCl, Shanghai Chemical Co., Shanghai, China), tris (hydroxymethyl)aminomethane (Tris), (Sigma), hydrochloric acid (Shenyang Chemical Co., Liaoning, China), λ-dsDNA (48 kb, Takara Biotechnology Co. Ltd., Dalian, China), ethidium bromide (EB) (Beijing BioDee Biotechnology Co. Ltd., Beijing, China), boric acid (H_3_BO_3_, Tianjin Bodi Chemical Co. Ltd., Tianjin, China), ethylenediaminetetraacetic acid (EDTA) (Shanghai Chemical Co., Shanghai, China). Buffers used in this work include Tris–HCl (pH 9.13), TBE (8.9 mM Tris, 0.2 mM EDTA and 8.9 mM H_3_BO_3_, pH 8.6). All aqueous solutions were prepared with Milli-Q water of 18.2 MΩ cm.

### 2.2. Instruments

The scanning electron microscope (SEM) picture was obtained with the SEM (SSX-550) from Shimatzu, Japan. The DC voltage supply unit was homemade (0–1500 V). Microscope fluorescent image was acquired with inverted fluorescent microscope (AE 31) from Motic, Xiamen, China, with a thermoelectrically cooled CCD camera (3000C), Image Advanced 3.2 software and 100 W dc Hg lamp. Electrochemical experiments were carried out using an electrochemical workstation (CHI 760B, Shanghai, China). An Ag/AgCl electrode was used in the rectification and translocation assays, and platinum electrodes were used for applying the voltage during the HF etching of the capillary.

### 2.3. Procedures

**Figure 1.**
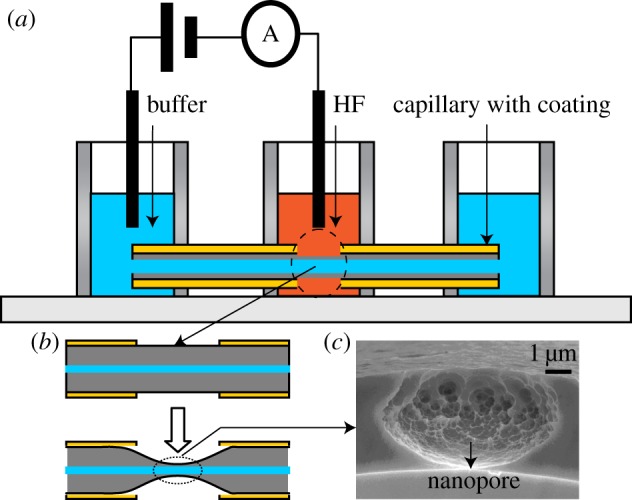
(*a*) Schematic of the single nanopore generation system. (*b*) Reduced wall thickness owing to HF etching. (*c*) Image of cross-sectional view of the micropore and the single nanopore on the inside wall of the capillary, as indicated by the arrow.

Fabrication of the nanofluidic interface was conducted following the procedure as described in [35], as schematically shown in figure 1. Briefly, approximately 1 mm of the polyimide coating of a piece of capillary (5 cm) was removed, and exposed the naked capillary wall to the HF etchant filled in the middle reservoir. A buffer solution 0.05 mol l^−1^ Tris–HCl was filled in the capillary from the end reservoirs. During etching of the outside wall of the capillary, a DC voltage was applied between the etching capillary wall, with the capillary inside positive. When the current jumped from the background level, the etchant was removed immediately and we rinsed the etching interface firstly with 2.5 mol l^−1^ NaOH, and then with water. Post overetching was performed by exposing the interface to the etchant for a given time without voltage bias.

For observation of the stacking fluorescent spot inside the capillary at the interface, a mixture of 1 µg ml^−1^ EB and 3.1 ng µl^−1^ dsDNA in 1× TBE solution was filled inside the capillary, and only the TBE buffer solution was filled in the middle reservoir. A DC voltage was applied with middle reservoir positive, and both the end reservoirs were grounded. A yellow fluorescent spot became visible under the microscope, and acquired by the CCD camera. Rectification and translocation assays were conducted with the electrochemical workstation by applying the voltage through two Ag/AgCl electrodes between the middle and end reservoirs, respectively.

## 3. Results and discussion

### 3.1. Scanning electron microscope characterization of the interface

**Figure 2.**
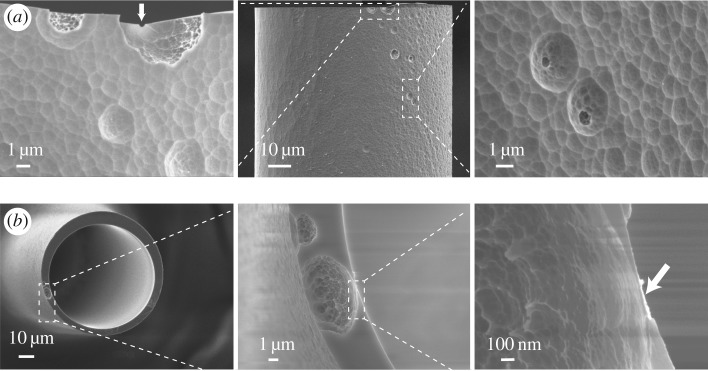
SEM pictures of the etched interface on the wall of the fused silica glass tube. (*a*) Top views; (*b*) cross-sectional views. Scale bars are as indicated in the respective SEM images. The sample was Au coated before SEM measurement.

To have a clear view of the pore(s), the etched interface was characterized by SEM, and a representative result is shown in figure 2. Normally, less than 50 V was applied to prepare an interface for ICP stacking purposes. To achieve a successful SEM sample with thicker wall and larger pores, this sample was prepared under 200 V bias. The etching stopped right after the monitoring current jumped, and the sample was broken for SEM observations of the surface and cross-sectional view of the etched interface. As can be seen from the SEM images of both the etched wall surface (top row) and the cross-section (bottom row), some pits were noted on the surface, and a bowl-shaped micropore (approx. 10 µm) across the thinned capillary wall (approx. 6 µm) was clearly seen. The single nanopore is located at the bottom of the cavity opening to the inside wall of the channel as indicated with arrows in the top-left and bottom-right SEM pictures. The pore is embedded in the bulk fused silica wall with gradually reduced thickness, which is beneficial for the strength of the pore. As can be seen from the SEM, beside the cross wall micropore, other pits not yet perforated are also seen. The outer surface is rough with pit features of microns as a result of HF etching of the glass surface [36]. This is the first clear SEM witness of the pores on the HF-etched fused silica capillary interface.

### 3.2. Principle of single nanopore generation

**Figure 3.**
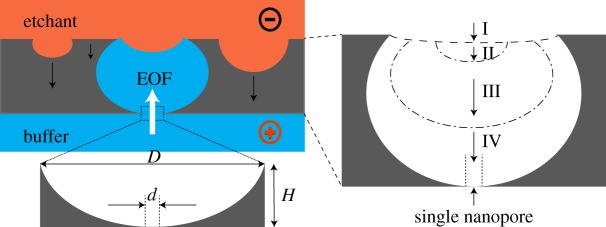
Schematic of stochastic development of the pits and the auto-stop etching of the perforated pore under DC voltage bias. The bottom insert is a schematic of the single nanopore with diameter of *d* opening to the inside of the wall. Right insert is schematic of pore development stages: (I) etched rough surface; (II) pre-pit; (III) cavity; (IV) perforation of a cavity to form the single nanopore at the bottom. EOF, electroosmosis flow.

Originally, the voltage was applied mainly for indicating the time to stop etching. We noted later that the polarity of this voltage was also important for the successful preparation of the ion conductive interface [33,35]. Based on the SEM observation as shown in figure 2, the number of pits is limited on the etched section of the outside wall, and the competitive perforation of the pits can be viewed as a stochastic event, as schematically shown in figure 3. It is well known that the probability that two or more independent events occur at the same time is very low. Therefore, most of the time, only one of the pits perforates at the time the interface becomes ion conductive. With the etchant side negative, electroosmotic flow towards outside of the capillary initiated as soon as the interface becomes conductive thanks to the existence of the negative charges on the fused silica surface. The etching in the cavity stopped automatically owing to the replacement of the etchant in the pore by the buffer from inside of the capillary, allowing more cleaning time to avoid further etching of the perforated cavity.

The applied voltage correlates with the resulted remaining wall thickness. When the interface became ion conductive, the remaining wall thickness was approximately 6 µm under 200 V bias, and only approximately 2 µm under 45 V. In both cases, the resulting electric field across the remaining wall was about 2–3 times greater than the dielectric breakdown of quartz of approximately 10 MV m^−1^ [37]. It is reasonable to attribute the pit perforation to a dielectric breakdown effect [29]. So, the process may be described by four stages: the roughed surface (stage I) developed into pits (stage II), and the pits into cavities (stage III) and one of the cavities eventually perforates by dielectric breakdown and results in only one single nanopore on the bottom of the cavity (stage IV). It should be noted that the pore size correlates with the voltage bias, for example, under 200 V the opening was about 300 nm as shown in figure 2. To achieve a smaller size single nanopore as estimated by ionic conductance measurement for nanofluidic effects, lower voltage is preferred, as presented in the following section.

### 3.3. Pore size control and estimation

The newly born interface was very reproducible, as indicated by the high reproducibility (relative standard deviation ∼ 5%) of the jumping current level [35], even under different HF concentrations (electronic supplementary material, figure S2A). The electrolyte conductance of the interface correlates with both the opening size and the pore shape [38,39]. So, the conductance can be used to estimate the pore size online without destroying the sample as in the case of SEM assay. Post overetching could enlarge the pore size. However, HF etching with or without voltage bias makes a difference. With a voltage bias, the current jumped from zero to approximately 8 µA and was maintained for a while before a second jump was observed (electronic supplementary material, figure S2A). The second jump should be ascribed to either more pits perforated or complete collapse of the etched section of the capillary, and thus actually short connected. Under the same voltage bias, the current level between the two jumps was less dependent on the etchant concentration, indicating that the pore size was mainly defined by the initial dielectric breakdown. However, the time gap between the two current jumps depends very much on the etchant concentration. It took more than 15 min with 5% HF to see the second jump, but only less than 3 min with 20% HF. With less concentrated HF, more time is allowed for the cleaning procedure.

To maintain the smallest size of the pore, the cleaning procedure should be initiated right after the first current jump. Nonetheless, the resulting pore may still be widened owing to continued etching by the residual HF during the cleaning procedure, especially when the etchant is highly concentrated. This provides a pore size tuning mechanism by continuously exposing the interface to the etchant, as can be seen from the pore resistance development of three post etched samples without voltage bias, as shown in the electronic supplementary material, figure S2B. The pore resistance was measured (refer to section S2 in the electronic supplementary material) after exposing the sample to the etchant for a given time. As expected, in all three cases, the pore resistance decreased quickly with time, indicating that the pore size was increasing. For an approximated model of the pore (refer to section S3 in the electronic supplementary material), pore resistance of 1 MΩ with 1 M KCl electrolyte corresponds to a pore size of about 56 nm. The estimated pore size ranges from 3 to 32 nm, as listed in the electronic supplementary material, table S1. The diversity of the pore resistance should be ascribed to the differences introduced in the manual cleaning operation. The pore resistance is mostly below 10 MΩ, which is much lower than that of the high aspect ratio glass pipette type channel [26], indicating its low aspect ratio nature.

### 3.4. Nanofluidic effects with this interface

**Figure 4.**
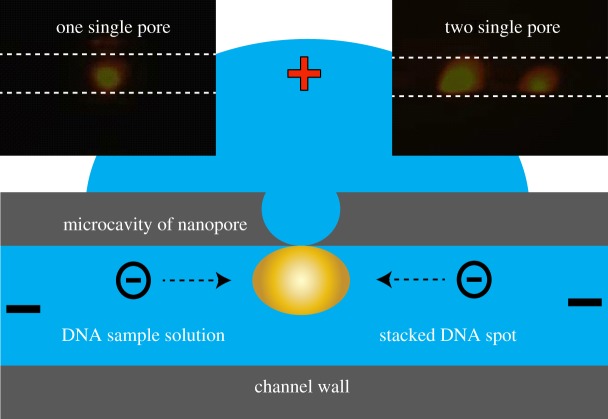
Localization of the single nanopore(s) by microscopic fluorescent imaging using ion concentration effect of dsDNA: 3.2 ng µl^−1^ *λ*DNA (48 kb) in pH 8.6 1× TBE with 1 µg ml^−1^ EB was filled inside the channel, and blank buffer solution outside of the capillary. Upon application of the 100 V with capillary inside negative, the labelled DNA migrate to the pore and stacked there and appeared as a fluorescent spot. Upper left CCD image is the case of one stacked spot, indicating the existence of only one single nanopore, and the upper right of two spots, indicating two nanopores occurred at the same time. The dotted lines indicate the inside diameter of the 100 µm i.d. capillary.

**Figure 5.**
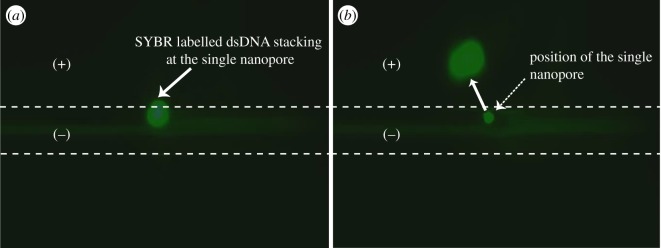
Electrokinetic stacking of dsDNA (*a*) and intermittent translocation of the stacking cluster through the single nanopore interface (*b*) as visualized by fluorescent microscope. Except that the EB is changed with 0.2× SYBR Green I, other conditions are the same as in figure 4.

With the above observations, we now realize that the electrokinetic stacking achieved with this type of interface [33–35] could have been the result of ICP locally with the single nanopore interface. As the ICP effect can be observed under a fluorescence microscope with fluorescent probe molecules, the number of pores may also be visually confirmed by a fluorescent microscope in addition to SEM and conductance characterizations. As shown in figure 4, when the outside is positively polarized, negatively charged fluorescent probe ions pre-loaded inside the capillary migrate to the interface and are locally stacked at the pore interface because of Donnan exclusion [35]. Thanks to the transparency of the glass substrate, this could be clearly observed under the fluorescent microscope with a 100 W DC Hg lamp and EB labelled dsDNA probe which emits yellow fluorescence under green light illumination. Initially, the diluted EB labelled dsDNA was invisible. In 50 s with 100 V bias, the stacked dsDNA appeared as one yellow spot in the dark background, as shown in the upper left insert. In the study of the ICP effect with this type of interface with dsDNA and other fluorescent probes, we noted that most of the time, only one stacking spot appeared on the interface. We indeed occasionally observed two stacking spots on the same interface, as shown in the upper right insert of figure 4. However, we have not seen a case of more than two spots on the same interface at the same time. This suggests that in most cases, truly ONE single nanopore was generated on the interface with the presented method. It is interesting to note that although dsDNA could be stacked at the interface for a long time (up to tens of minutes), we did observe occasional translocation of the stacking band through the interface, as shown in figure 5. The dsDNA was labelled with SYBR Green I in this assay. Under the voltage bias, the stacking cluster vibrated constantly during the stacking process, and sometimes translocated through the interface towards the cathode. A new stacking spot soon developed again at the interface after the event. We assume that the translocation may be owing to the transient breakdown of the Donnan exclusion by the turbulence effect [40].

**Figure 6.**
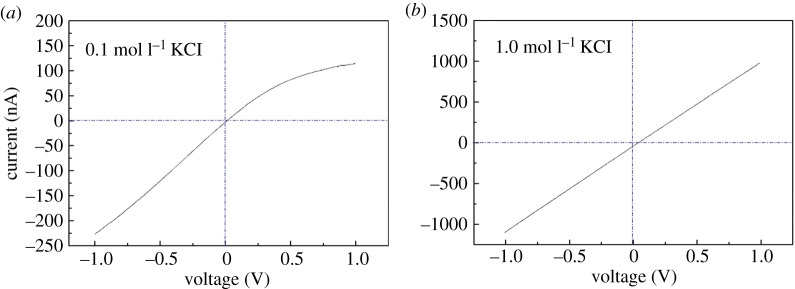
*I*–*V* curves of the single nanopore interface. (*a*) 0.1 M KCl; (*b*) 1.0 M KCl. Working electrode positioned in the middle reservoir as shown in figure 1, scan from −1.0 V to +1.0 V at the rate of 50 mV s^−1^ AgCl/Ag electrodes. The pore size is about 10 nm as estimated by the conductivity measurement and the model as described in the electronic supplementary material.

Besides the above-mentioned ICP effect, ion rectification should also be expected with this single nanopore interface, because the pore is asymmetrical and with a charged surface. As can be seen in figure 6*a*, the curve obviously deviates from linearity with 0.1 M KCl, and showed a rectification ratio of 2 at ±1.0 V. Both the curve pattern and direction of rectification agree well with the geometry of the interface, and are consistent with a negatively charged conical nanopore [41]. However, with 1.0 M KCl, the curves are linear, as shown in figure 6*b*.

**Figure 7.**
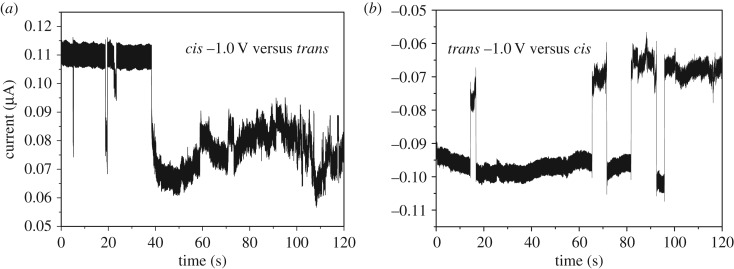
Translocation of dsDNA through the single nanopore. (*a*) DNA was initially loaded in the middle reservoir as shown in figure 1; (*b*) result of reversing the voltage polarity. 0.2 nM *λ* DNA in 1× TBE 0.1 KCl media, Ag/AgCl electrodes, acquisition rate 1 kHz.

The current development under given voltage bias was also monitored when non-labelled dsDNA in 1× TBE with 0.1 M KCl was loaded in the middle reservoir (defined as the *cis* side) and the preliminary results are shown in figure 7. Some negative current pulses were noted before the current became instable at a lower current level. When the polarity was reversed, the current returned to the base level, but soon was back to a lower level with some longer current spikes. The lower current state could have been the result of ion depletion caused by the ICP effect, and the pulses may be ascribed to the translocation of the dsDNA probes or their stacking clusters. No pulses were noted when lower voltage was applied, probably owing to the negatively charged probe that could not overcome the Donnan exclusion barrier. Further investigation will be conducted and reported in another topic.

## 4. Conclusion

In summary, we achieved ONE single solid-stage nanopore on the wall of fused silica capillary by electric field-assisted HF etching without cleaning room conditions and a capillary pull machine. The voltage applied during HF etching not only helps to indicate the perforation and stops the etching automatically, but also assists the formation of the cavities and final perforation of a cavity by dielectric breakdown. Unlike a pipette tip or conical pore at the terminal of a glass tube, the single nanopore thus generated on the thinned wall is characterized in a bowl shape and thin edge, corresponding to low aspect ratio. This method is simple and cost-effective, and exhibits the major features of a nanofluidic interface, such as ICP, rectification and translocation. Nanofluidic assays relevant with a single nanopore could be performed with this new platform affordable to common laboratories.

## Supplementary Material

Supporting information and data

## Data accessibility

Electronic supplementary material is available with this paper.
