# Supplementary material for: Making of a single solid-state nanopore on the wall of fused silica capillary
Source: R Soc Open Sci. 2018 Jun 6;5(6):171633. doi: 10.1098/rsos.171633 (PMC6030265; doi:10.1098/rsos.171633)
Supplement: Supporting information and data [file rsos171633supp1.doc]

Supporting Information

Making of a Single Solid-State Nanopore on the Wall

of Fused Silica Capillary

Fang Fang, Yan-Qin He, Li Tian, Yun-Yun Li, Zhi-Yong Wu*

Research Center for Analytical Sciences, Department of Chemistry,

College of Sciences, Northeastern University, Shenyang, 110819, China.

1. **Figures**


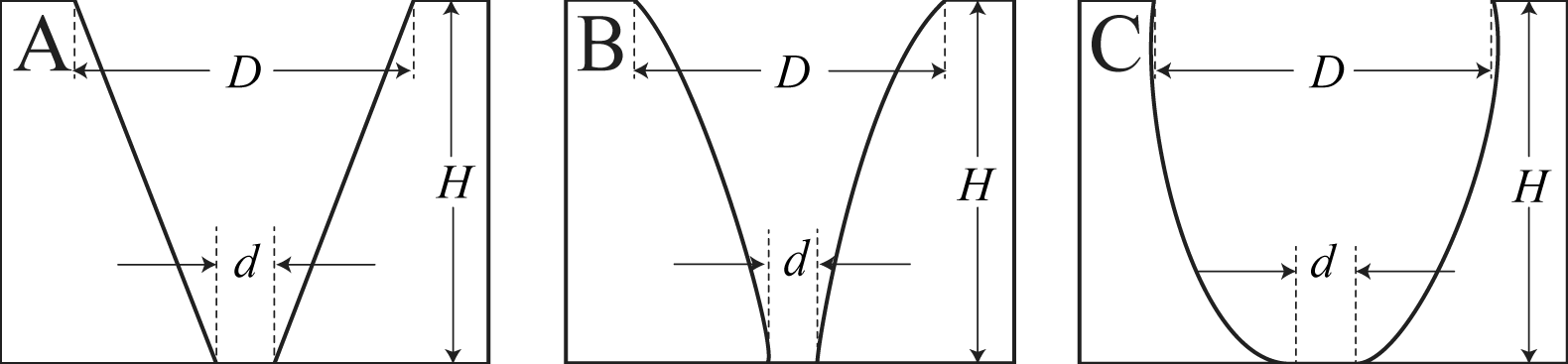


Figure S1. Schematic geometry of 3 typical single solid nanopores (cross sectional view). A-conical, B-pipette tip, C-bowl shape. The main parameters to define a pore include the substrate thickness (H), open side diameter (D) and narrow side diameter (d). The feature size of a nanopore is represented by d, supposing the pore is coaxially symmetrical.


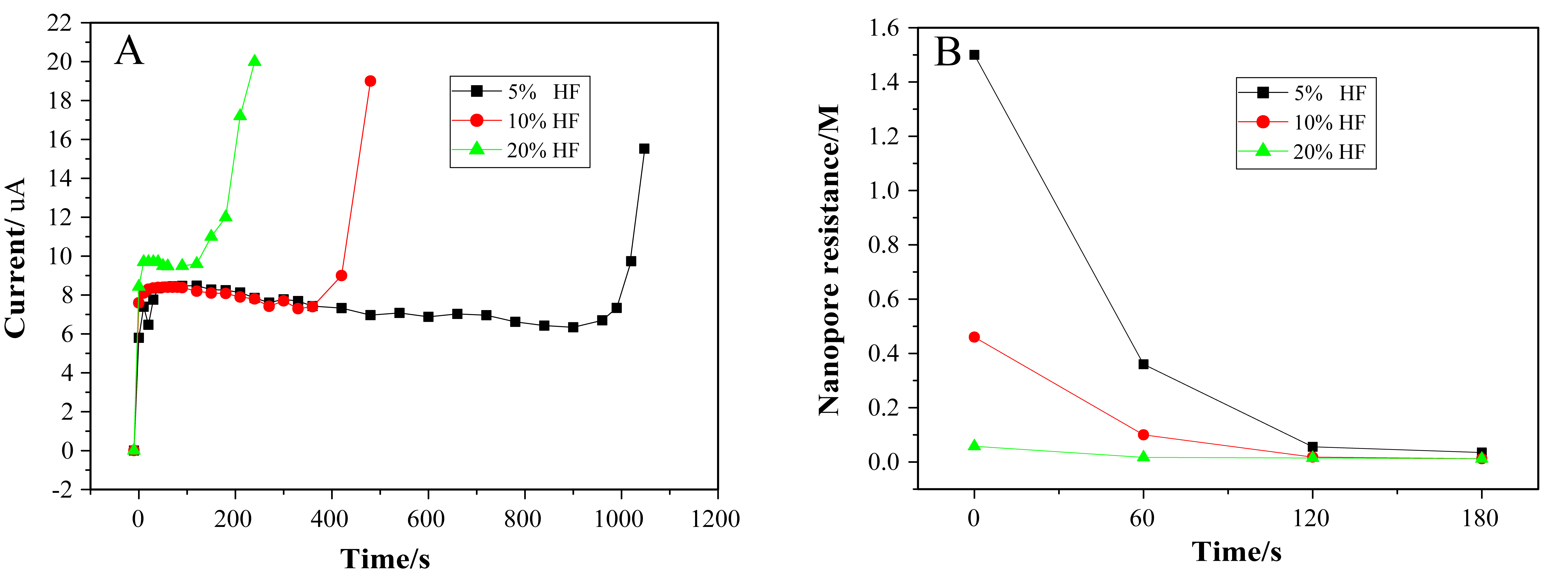


Figure S2. A: current development by time after initial current jump with 20 V voltage bias, 50 mM pH 9.0 Tris-HCl with 0.1 M KCl. B: Pore resistance (with 1 M KCl) development by post over etching time without voltage bias.

1. **Nanochannel resistance**

Resistance measurement of the ion conductive interface was conducted the same manner as described in [Microfluid Nanofluid 2013, 14(1-2): 69-76]. First we measure the resistance of the interface and half of the capillary by applying a voltage through one end reservoir and that of the middle, and then that of the total capillary channel by applying a voltage across the two end reservoirs. The equivalent circuit is shown in Figure S2, and we have the relations (1), (2) and (3).

$R_{1\text{i}}=R_{1}+R_{\text{i}}$ (1)

$R_{\text{2i}}=R_{2}+R_{\text{i}}$ (2)

$R_{12}=R_{1}+R_{2}$ (3)

R1 and R2 refer to the resistance of the left and right half of the capillary, and R12 the sum of the whole capillary. With the measured R1i, R2i and R12, the resistance of the interface Ri can be calculated according to (4).

$R{}_{\text{i}}=\frac{R_{1\text{i}}+R_{2\text{i}}-R_{12}}{2}$ (4)

The interface resistance (Ri) can be viewed as serially connected to the micropore (Rm), and the nanopore (Rn), as in (5).

$R_{\text{i}}=R_{\text{m}}+R_{\text{n}}$ (5)


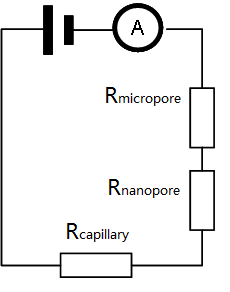


Figure S3. Equivalent circuit of the loop

1. **Single nanopore model analysis**


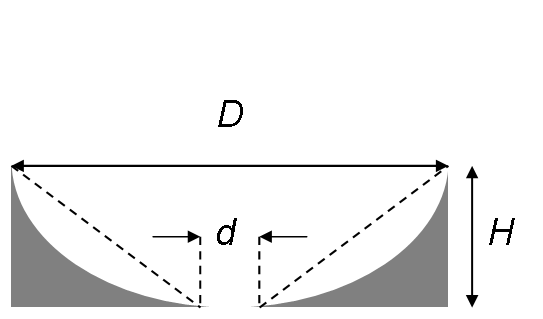


Figure S4. Approximation of the nanopore

Suppose a approximated cylindrical model of the nanopore, the pore diameter d can be estimated by the pore resistance with equation (6).

$\text{d}=2\sqrt{\frac{\text{H}}{k\cdot\pi\cdot\text{R}}}$ (6)

H is the thickness, k the conductivity of the electrolyte, and R the pore resistance. The resistance of a cylindrical cavity of 3 μm depth and radius 3 μm filled with 1 M KCl is 10 kΩ, which is much smaller than the measured resistance as listed in Table S1. So the interface can be viewed as a macrocavity and nanopore connected in series, and the total resistance is mainly defined by the thin edge nanopore. Since the nanopore is a result of electrical breakdown of a sub-pits in the microcavity, the shape of the nanopore should also a sub-fracture of the microcavity, thus the pore is more like a conical than a cylindrical shape. For a conical pore with thickness (H) and wider side diameter (D), narrow side diameter d is defined by equation (7) [Appl. Phys. A 76, 2003, 781-785]

$\text{d}=\frac{\text{4H}}{k\cdot\pi\cdot\text{R}\cdot\text{D}}$ (7)

With approximation of the pore thickness H is the same as the pore outer radius D/2, we have equation (8), with which the inner pore size d can be roughly estimated.

$\text{d}\approx\frac{2}{k\cdot\pi\cdot\text{R}}$ (8)

With this equation, we may estimate that with 1 M KCl electrolyte, 1 MΩ pore resistance corresponds to a d of 56 nm. Table S1 lists 28 samples with estimated d range from 3.2 to 34.6 nm.

**Table S1. The estimated resistance (MΩ**) and diameter (nm) of the nanopore

| No. sample | R1i | R2i | R12 | Ri | Estimated d/nm |
| --- | --- | --- | --- | --- | --- |
| 1 | 1.98 | 1.88 | 0.62 | 1.62 | 34.6 |
| 2 | 2.92 | 2.45 | 0.58 | 2.40 | 23.4 |
| 3 | 3.42 | 3.70 | 0.61 | 3.26 | 17.2 |
| 4 | 5.86 | 4.37 | 0.63 | 4.80 | 11.6 |
| 5 | 3.56 | 3.42 | 0.63 | 3.18 | 17.7 |
| 6 | 8.79 | 9.87 | 0.67 | 8.99 | 6.2 |
| 7 | 1.98 | 2.29 | 0.69 | 1.79 | 31.2 |
| 8 | 2.95 | 3.00 | 0.69 | 2.63 | 21.2 |
| 9 | 10.38 | 8.08 | 0.69 | 8.88 | 3.2 |
| 10 | 3.16 | 3.07 | 0.73 | 2.75 | 20.4 |
| 11# | 9.87 | 7.85 | 1.39 | 8.16 | 6.8 |
| 12# | 4.51 | 5.54 | 1.26 | 4.40 | 12.8 |
| 13# | 11.89 | 9.70 | 1.21 | 10.18 | 5.6 |
| 14 | 3.61 | 5.46 | 0.72 | 4.17 | 13.4 |
| 15 | 3.03 | 2.62 | 0.75 | 2.45 | 22.8 |
| 16 | 4.01 | 3.80 | 0.69 | 3.56 | 15.8 |
| 17 | 3.59 | 2.58 | 0.69 | 2.75 | 20.4 |
| 18 | 9.06 | 6.67 | 0.63 | 7.55 | 7.4 |
| 19 | 10.76 | 12.80 | 0.62 | 11.47 | 4.8 |
| 20 | 8.15 | 9.20 | 0.65 | 8.35 | 6.8 |
| 21 | 4.41 | 4.98 | 0.61 | 4.39 | 12.8 |
| 22 | 2.13 | 2.66 | 0.62 | 2.08 | 27.0 |
| 23 | 7.45 | 6.61 | 0.64 | 6.71 | 8.4 |
| 24 | 3.54 | 4.70 | 0.63 | 3.80 | 14.8 |
| 25 | 3.83 | 3.87 | 0.58 | 3.56 | 15.8 |
| 26 | 2.75 | 3.89 | 0.60 | 3.02 | 18.6 |
| 27 | 2.72 | 2.53 | 0.60 | 2.32 | 24.2 |
| 28 | 2.74 | 2.60 | 0.58 | 2.38 | 23.6 |

Note: 1. # by capillaries of 75 μm id, and all others 100 μm id.; 2. measurement was conducted at room temperature, the conductivity of 1 M KCl was taken as 0.1118 S/cm. All sample was etched with 5 % HF (w/v) with 45 V bias.
